# Correlation between serum levels of cyclooxygenase-2, aquaporin-1, pepsinogen I, gastrin-17 and pathological features of Laryngeal Cancer

**DOI:** 10.12669/pjms.40.11.10417

**Published:** 2024-12

**Authors:** Danping Li, Xiaoqiong Qian, Cong Li, Song Shi

**Affiliations:** 1 Danping Li, Department of Otorhinolaryngology, Tongren Hospital, Shanghai Jiao Tong University School of Medicine, 111 Xianxia Road, Shanghai, 200336, P.R. China; 2 Xiaoqiong Qian, Department of Otorhinolaryngology, Tongren Hospital, Shanghai Jiao Tong University School of Medicine, 111 Xianxia Road, Shanghai, 200336, P.R. China; 3 Cong Li, Department of Otorhinolaryngology, Tongren Hospital, Shanghai Jiao Tong University School of Medicine, 111 Xianxia Road, Shanghai, 200336, P.R. China; 4 Song Shi, Department of Otorhinolaryngology, Tongren Hospital, Shanghai Jiao Tong University School of Medicine, 111 Xianxia Road, Shanghai, 200336, P.R. China

**Keywords:** Cyclooxygenase-2, Aquaporin-1, Pepsinogen I, Gastrin-17, Laryngeal cancer

## Abstract

**Objective:**

To explore the association between the levels of cyclooxygenase-2 (COX-2), aquaporin-1 (AQP1), pepsinogen I (PGI), and gastrin-17 (G17) and pathological features of laryngeal cancer (LC).

**Methods:**

Clinical data of 120 patients with LC, treated in Shanghai Jiao Tong University School of Medicine from May 2022 to May 2023, as well as 120 healthy individuals who underwent physical examinations during the same period, were retrospectively analyzed. Serum levels of COX-2, AQP1, PGI, and G17 of LC patients and the healthy cohort were compared, and their association with pathological features of LC was investigated.

**Results:**

LC was associated with significantly higher serum levels of COX-2, AQP1, and G17 and lower of PGI compared to the control group (*P*<0.05). Levels of COX-2, AQP1, and G17 positively correlated with the tumor, node and metastasis (TNM) staging, infiltration depth, and the lymph node metastases, and negatively correlated with the degree of differentiation (*P*<0.05). There was a significant negative correlation between the level of PGI and TNM staging, infiltration depth, and lymph node metastases. PG1 also positively correlated with the degree of differentiation (*P*<0.05). Area under curve (AUC) of combined COX-2, AQP1, PG-I, and G17 was significantly higher than that of each index alone (*P*<0.05).

**Conclusions:**

Levels of COX-2, AQP1, PGI, and G17 showed significant association with specific pathological features of LC. Serum levels of COX-2, AQP1, PGI, and G17 have certain predictive value for LC, and the combined predictive value of these four indicators is higher than that of a single indicator.

## INTRODUCTION

Laryngeal cancer (LC) is a common malignant tumor of head and neck, and its incidence is on the rise.^1^ Due to the lack of obvious early symptoms, many patients are diagnosed with LC at the late stages, which significantly affects treatment outcome and patient’s prognosis.^1^,^2^ Therefore, identifying reliable biomarkers to assist in the early diagnosis of LC has become one of the focuses of LC research.^2^,^3^ Additionally, such biomarkers may provide important information about disease development, treatment response, and prognosis prediction.^3^,^4^

However, there is currently a lack of well-defined biomarkers for clinical application in LC.^5^ Previous research has identified several proteins that may serve as such biomarkers. Cyclooxygenase-2 (COX-2), an enzyme that plays a role in regulating inflammation and cell proliferation in normal physiological processes,^6^ is specifically associated with poorer prognosis of LC.^7^ Aquaporin-1 (AQP1), a transmembrane protein that forms water channels on the cell membrane and is responsible for regulating the balance of water inside and outside the cell,^8^ has been reported to be overexpressed in LC cells.^9^ Another such potential biomarker is pepsinogen I (PGI), a proteasogen produced by gastric mucosal cells, which is converted into pepsin,^10^ which is associated with LC development.^11^ Gastrin-17 (G17), a hormone produced in gastric mucosal cells, participates in the regulation of gastric acid secretion and the proliferation of gastric mucosal cells,^12^ and showed a good sensitivity and specificity for screening of both early and progressive gastric cancer.^13^ There are few studies exploring the association between G17 and LC, but Zhang et al^14^ showed that G17 is of great significance for the diagnosis and differentiation of early laryngeal cancer. Thus, this study aimed to explore the correlation between serum COX-2, AQP1, PGI, and G17 and pathological features of LC to evaluate the clinical potential of these indicators and their combined applications in patients with LC and to provide more evidence for existing studies.

## METHODS

Clinical data of LC patients, diagnosed and treated in Shanghai Jiao Tong University School of Medicine from May 2022 to May 2023 (the LC group), and healthy examinees from the same hospital during the same period (the control group). A total of 250 healthy individuals from the same period were screed and 31 individuals were excluded. Gender and age of the patients were matched 1:1, and finally 120 patients were included in each group.

### Inclusion criteria:

The patients were aged over 18 years, serum COX-2, AQP1, PGI, and G17 levels were measured, clinical data were complete, and patients in the LC group were diagnosed with LC.

### Exclusion criteria:

Patients with significant organic heart disease, hepatic or renal dysfunction, other vital organ diseases, or other malignant tumors, and pregnant or lactating women. A total of 249 patients were screened based on the inclusion and exclusion criteria, and 47 patients were excluded.

### Ethical Approval:

The ethics committee of our hospital approved this study 2023-007, Date: February 23^rd^, 2023.

### Information collection:

Baseline data: gender, age, BMI, and educational level of the patients were collected.

Serum levels of COX-2, AQP1, PGI, and G17: 3 ml of venous blood sample were collected from the patients in the fasting state in the morning, and the serum was separated by centrifugation at 1500 r/min for 15 min for COX-2, 4000 r/min for 10 min for AQP1, and 2000 r/min for 15 min for PGI and G17, and then were kept at -20°C for later measurement. Measurements were conducted by enzyme-linked immunosorbent assay (***ELISA***) using the reagent kit is by BlueGene company according to the manufacturer’s instructions. All procedures were performed by the same group of experienced physicians.

### Pathological features:

Based on the results of the pathological examination, TNM staging was performed according to the 2002 American Joint Commission on Cancer (AJCC), including stages I, II, III, and IV. T staging was performed according to the depth of infiltration, including T1, T2, T3, and T4 stages. Lymph node metastasis was divided into three types: N0, N1, and N2. Differentiation degree was divided into three categories: highly differentiated, moderately differentiated, and poorly differentiated.

### Statistical analysis:

All data analysis was conducted using SPSS 26.0 software (IBM Corp, Armonk, NY, USA). Quantitative data were expressed as mean ± standard deviation, and independent sample t-test was used for inter group comparison. Count data were analyzed using chi square test. Spearman correlation analysis was used to evaluate the association between serum COX-2, AQP1, PGI and G17 levels and different pathological features of LC. Receiver operating characteristic (ROC) curves was used to analyze serum levels of COX-2, AQP1, PGI, and G17, as well as their combined predictive value for LC. *P*<0.05 indicates a statistically significant difference.

## RESULTS

There was no statistically significant difference in the baseline data such as gender, age, BMI, and educational level between the two groups (*P*>0.05) (Table-I). Serum levels of COX-2, AQP1, and G17 in the LC group were higher than those in the control group (*P*<0.05), while serum PGI levels were lower in the LC group than those in the control group (*P*<0.05) (Table-II). Patients with more advanced TNM stage, infiltration depth, and lymph node metastasis had increased serum COX-2, AQP1, and G-17 levels and decreased PG-I levels (*P*<0.05). In addition, the higher the degree of differentiation, the lower the serum COX-2, AQP1, and G-17 levels, and the higher the level of PG-I (*P*<0.05). (Table-III). Spearman correlation analysis showed that serum levels of COX-2, AQP1, and G17 were significantly positively correlated with TNM staging, infiltration depth, and lymph node metastasis (*P*<0.05), and significantly negatively correlated with the degree of differentiation (*P*<0.05). In contrast, serum level of PGI significantly negatively correlated with TNM staging, infiltration depth, and lymph node metastasis (*P*<0.05), and significantly positively correlated with the degree of differentiation (*P*<0.05) (Table-IV).

**Table-I:** Comparison of baseline data between the two groups.

Group	n	Gender		Age (years))	BMI (kg/m^2^)	Educational level		
	
		Male	Female			Junior high school and below	High school and secondary school	College or above
Case group	120	53 (44.17)	67 (55.83)	52.68±4.29	24.71±2.28	46 (38.33)	31 (25.83)	43 (35.83)
Control group	120	49 (40.83)	71 (59.17)	53.03±4.34	24.75±2.53	49 (40.83)	34 (28.33)	37 (30.83)
*χ* ^2^ */t*	-	0.273		-0.628	-0.123	0.683		
*P*	-	0.601		0.530	0.902	0.711		

**Table-II:** Comparison of serum COX-2, AQP1, PGI and G17 levels between the two groups.

Group	n	COX-2	AQP1	PGI	G17
Case group	120	71.48±13.59	39.63±11.04	69.74±15.82	13.79±3.29
Control group	120	57.59±9.06	24.32±3.01	89.72±17.05	8.37±1.94
*t*	-	9.314	14.657	-9.409	15.546
*P*	-	<0.001	<0.001	<0.001	<0.001

**Table-III:** Serum COX-2, AQP1, PGI and G17 levels of different pathological features in patients with laryngeal cancer.

Pathological feature	Sort	n	COX-2	AQP1 (ng/mL)	PGI (ug/L)	G17 (pmoL/L)
TMN staging	I	12	59.78±9.51	26.37±3.12	86.71±16.87	9.82±2.06
	II	56	67.90±10.29	33.39±6.34	75.40±13.52	12.75±2.48
	III	45	76.61±13.74	48.35±7.49	61.18±10.53	15.39±2.67
	IV	7	87.22±16.27	56.23±3.70	50.40±9.52	18.74±2.98
	F	-	12.205	76.504	23.199	27.562
	P	-	<0.001	<0.001	<0.001	<0.001
Degree of differentiation	Poorly differentiated	37	81.47±13.60	50.13±8.03	61.85±11.42	16.33±3.20
	Moderately differentiated	57	68.42±11.55	37.17±9.02	71.91±15.93	13.24±2.47
	Highly differentiated	26	63.98±9.34	30.10±5.81	76.23±16.99	11.39±2.62
	F	-	20.412	51.366	8.203	26.920
	P	-	<0.001	<0.001	<0.001	<0.001
Depth of infiltration	T1	20	63.51±9.29	29.78±5.37	76.66±16.73	11.43±2.77
	T2	63	68.72±11.31	37.25±9.45	71.22±15.47	13.28±2.73
	T3	28	78.36±13.98	47.22±9.35	65.90±14.11	15.12±2.87
	T4	9	87.11±15.31	54.58±4.18	56.03±11.42	18.54±3.13
	F	-	12.193	26.662	4.657	16.204
	P	-	<0.001	<0.001	<0.001	<0.001
Lymph node metastasis	N0	25	63.54±7.65	30.88±6.18	77.36±18.95	11.42±2.78
	N1	61	70.29±12.77	38.16±10.38	71.79±14.85	13.47±2.62
	N2	34	79.46±14.53	48.70±8.33	60.47±10.02	16.13±3.29
	F	-	12.336	29.278	10.763	20.426
	P	-	<0.001	<0.001	<0.001	<0.001

**Table-IV:** Correlation analysis of serum COX-2, AQP1, PGI and G17 levels with different pathological features of patients with laryngeal cancer.

Variable	Coefficient	TNM staging	Degree of differentiation	Depth of infiltration	Lymph node metastasis
COX-2	*r_s_*	0.473	-0.482	0.453	0.412
	*p*	<0.001	<0.001	<0.001	<0.001
AQP1	*r_s_*	0.816	-0.676	0.63	0.575
	*p*	<0.001	<0.001	<0.001	<0.001
PGI	*r_s_*	-0.615	0.355	-0.332	-0.368
	*p*	<0.001	<0.001	<0.001	<0.001
G17	*r_s_*	0.618	-0.554	0.495	0.492
	*p*	<0.001	<0.001	<0.001	<0.001

**Fig.1:**
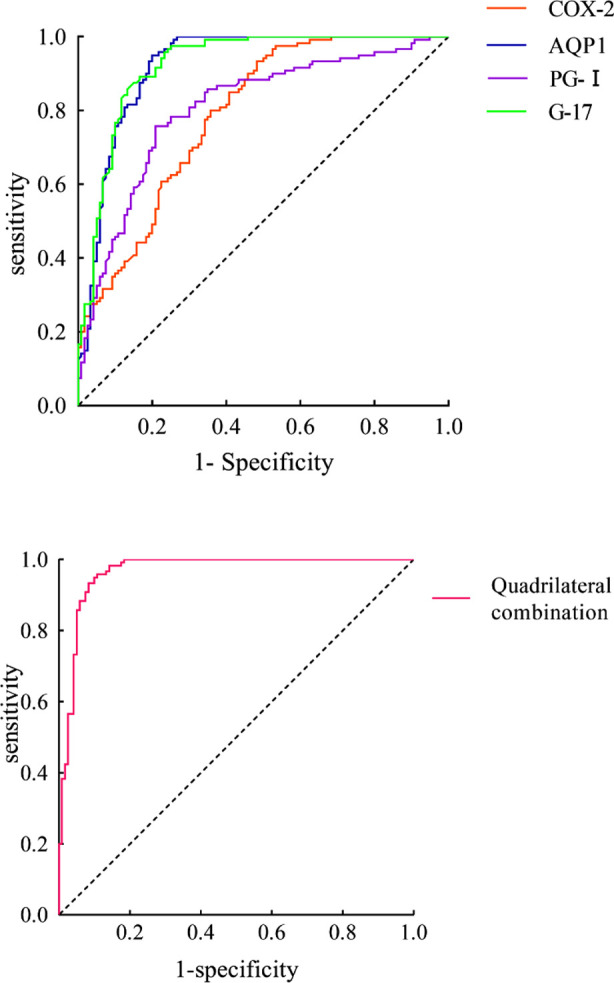
Serum COX-2, AQP1, PGI and G17 levels and ROC curve of combined prediction of laryngeal cancer.

In terms of predictive value for LC, the ROC curves showed that the area under curve (AUC) of COX-2, AQP1, PGI, G17, and the combined four indicators were 0.789, 0.922, 0.807, 0.924, and 0.967, respectively, indicating that they have certain predictive value for LC. When the cut-off value was defined, serum levels of COX-2, AQP1, PGI, and G17, as well as the corresponding sensitivity for combined prediction, were 0.775, 0.867, 0.808, 0.875, and 0.950, respectively, and the specificity was 0.608, 0.817, 0.692, 0.842, and 0.825, respectively. It is also showed that the combined AUC was higher than that of individual serum COX-2, AQP1, PGI, and G17 levels (*P*<0.05). Table-V, Fig.1

**Table-V:** Serum COX-2, AQP1, PGI and G17 levels and ROC analysis of combined prediction of laryngeal cancer.

Test result variable	AUC	SE	P	95%CI	cut-off value	Jorden index	sensitivity	specificity
COX-2	0.789	0.029	<0.001	0.733~0.845	59.85	0.383	0.775	0.608
AQP1	0.922	0.018	<0.001	0.886~0.958	27.205	0.684	0.867	0.817
PGI	0.807	0.029	<0.001	0.751~0.863	82.725	0.500	0.808	0.692
G17	0.924	0.018	<0.001	0.889~0.958	10.195	0.717	0.875	0.842
Quadrilateral combination	0.967	0.012	<0.001	0.944~0.989	0.7976	0.775	0.950	0.825

## DISCUSSION

The results of this study showed that serum levels of COX-2, AQP1, and G17 in the serum of patients with LC were higher than those in the control group, while the levels of PGI were lower. This indicates that COX-2, AQP1, and G17 may be overexpressed in LC patients, while the expression of PGI may be suppressed. As the severity of the disease (as indicated by TNM staging, infiltration depth, lymph node metastases) increases, levels of COX-2, AQP1, and G17 in the serum of LC patients also show an upward trend, while the levels of PGI decrease.^5^,^10^,^12^ This suggests that high levels of COX-2, AQP1, and G17 may be associated with the of LC, while low levels of PGI may reflect the invasiveness of the tumor.^15^ This is consistent with the findings of Zhang et al^14^ Malespín-Bendaña et al.^16^, Du et al.^7^, and Zubčić et al.^17^ Moreover, the current study also found that patients with highly differentiated LC had lower levels of COX-2, AQP1, and G17 in their serum, while higher levels of PGI were observed. This suggests that the expression of COX-2, AQP1, and G17 may be related to the differentiation status of LC, while high levels of PGI may be associated with better degree of differentiation.

We speculate that COX-2 is an inflammation related enzyme with relatively low expression under normal circumstances. It mainly participates in maintaining normal physiological state of the inflammatory response and tissue repair process.^5^,^18^ However, overexpression of COX-2 has been widely observed during tumor occurrence and metastasis.^6^,^18^ COX-2 participates in various signaling pathways of inflammation, proliferation, invasion, and angiogenesis by producing a class of bioactive substances called prostaglandins.^19^ These processes are crucial for the growth, diffusion, and metastasis of tumors.^18^,^19^ Therefore, high expression of COX-2 is associated with the occurrence, progression, and poor prognosis of tumors.^6^,^18^,^19^ AQP1 is a transmembrane protein primarily involved in the transport of intracellular and extracellular water molecules.^8^

In tumors, the abnormal expression of AQP1 is closely related to angiogenesis and the invasive ability of tumor cells.^20^ AQP1 can promote the absorption and excretion of water and other small molecules by tumor cells, thereby maintaining their osmotic balance and metabolic needs.^9^,^20^ G17 is a type of gastrin, which is mainly produced by normal gastric cells and participates in the regulation of gastric acid secretion.^10^ However, studies have reported high levels of G17 expression in LC, which may be related to abnormal activation of the gastrin system.^21^

The abnormal activation of the gastrin system may involve various factors, such as neural regulation imbalance, inflammatory response, and changes in the tumor microenvironment,^21^,^22^ and may lead to an increase in gastric acid secretion and the activation of other signaling pathways that promote tumor development.^22^ Therefore, high levels of G17 may be associated with the occurrence and progression of LC.^10^,^14^,^22^ Taken together, the abnormal expression of these biomarkers may be the result of cellular signaling pathway dysregulation and gene variation during the development of LC.^9^,^16^,^18^,^20^-^22^ The progression and deterioration of LC can lead to the proliferation, infiltration, and metastasis of tumor cells.^23^ These processes may be accompanied by intensified inflammatory response and increased angiogenesis, leading to upregulation of COX-2, AQP1, and G17 expression.^9^,^18^-^23^ We noted a correlation between the level of PGI and different pathological features. Usually, level of PGI is high, which may be related to normal digestive function.^24^,^25^

However, our results showed that in LC patients, serum level of PGI was significantly negatively correlated with TNM staging, infiltration depth, and lymph node metastasis, and significantly positively correlated with differentiation degree. The abnormal expression of PGI in LC patients may be related to changes in tumor progression and differentiation status. The specific mechanism may involve complex regulation of multiple factors, including inflammatory response, cell proliferation, and differentiation.^26^ The degree of LC differentiation may reflect functional status of tumor cells, and highly differentiated tumor cells may have better function and tissue structure.^27^ Therefore, they may exhibit lower levels of COX-2, AQP1, and G17, as well as higher levels of PGI.^24^-^27^

This study also showed that serum levels of COX-2, AQP1, PGI, and G17, as well as their combination, have high predictive value in LC, with AUCs of 0.789, 0.922, 0.807, 0.924, and 0.967, respectively. This indicates that these biomarkers have a certain predictive ability in distinguishing between LC and non-LC patients. The joint prediction of AUC values was significantly higher than that of serum COX-2, AQP1, PGI, and G17 levels alone. This indicates that by combining multiple biomarkers, clinical value of the model can be increased, capturing more variations and abnormal signals that may be related to LC. The combined use of indicators can improve the accuracy and reliability of predicting LC.

### Limitations:

This is a retrospective study with a small sample size. Additionally, only limited number of relevant indicators were studied. Therefore, our conclusions may have a certain degree of bias and further prospective multicenter study may be required to validate our results.

## CONCLUSION

This study shows a correlation between serum levels of COX-2, AQP1, PGI, and G17 and different pathological features in patients with LC. Serum levels of COX-2, AQP1, PGI, and G17 have certain predictive value for LC, and the combined predictive value of these four indicators is higher than that of a single indicator.

### Authors’ contributions:

**DL:** Conceived and designed the study, manuscript writing.

**XQ**, **CL** and **SS:** Collected the data and performed the analysis.

All authors have read, approved the final manuscript and are responsible for the integrity of the study.
